# A Functional rs353293 Polymorphism in the Promoter of miR-143/145 Is Associated with a Reduced Risk of Bladder Cancer

**DOI:** 10.1371/journal.pone.0159115

**Published:** 2016-07-20

**Authors:** Jun Wu, Qun Huang, Dongdong Meng, Minyu Huang, Chaowen Li, Tianzi Qin

**Affiliations:** Department of Urinary Surgery, Affiliated Hospital of Youjiang Medical College for Nationalities, Baise, 533000, Guangxi, China; University of Navarra, SPAIN

## Abstract

**Background and Objectives:**

MicroRNA (miR)-143/145, known as tumor suppressors, can promote cell apoptosis and differentiation, and suppress cell proliferation, invasion and migration. We performed a case-control study to investigate the association of rs353293 in the promoter region of miR-143/145 with bladder cancer risk.

**Methods:**

In total, 869 subjects including 333 cases and 536 controls were enrolled in this study, and the rs353293 polymorphism was genotyped by using a Taqman assay. The promoter activity was measured by the Dual-Luciferase Assay System.

**Results:**

We calculated an adjusted odds ratio of 0.64 for the presence of either AA/AG genotypes (95% CI 0.46–0.90) and 0.64 (95% CI 0.47–0.87) for carrying at least one A allele in bladder cancer. Stratified analyses showed that the AA/AG genotypes and the A allele were less prevalent in patients with low grade tumors, compared to those harboring higher grade bladder cancers (adjusted OR = 0.53, 95% CI, 0.30–0.94, *P* = 0.03 and adjusted OR = 0.54, 95% CI, 0.32–0.92, *P* = 0.02, respectively). *In vitro* luciferase reporter analysis showed that rs353293A allele had a lower activity compared with the rs353293G allele (*P* < 0.001).

**Conclusion:**

These findings suggest that the functional rs353293 polymorphism may be a useful biomarker to predict the risk of bladder cancer.

## Introduction

Bladder cancer (BC) is a malignancy arising from the urothelium of the urinary bladder. Globally, there are about 429,800 newly diagnosed cases in 2012 [1]. Although the stage-specific 5-year relative survival rate is 96% in the United States, there are an estimated 165,100 deaths occurred in 2012 worldwide [1,2]. Epidemiological studies have identified some risk factors for BC, such as tobacco smoking, occupational exposures to industrial chemicals, and dietary nitrates and arsenic [3–7]. Despite the falling number of smokers in the United States, the incidence rates and death rates have been stable over the last 10 years (www.seer.cancer.gov). Furthermore, a familial aggregation of urothelial cell carcinoma (UCC) was observed with an almost 2-fold increased risk among first-degree relatives of UCC patients [8], indicating that genetic factors are of great importance in the development of BC.

miRNAs are endogenous ~22 nt non-coding RNAs that play key regulatory roles by binding to the 3’ untranslated region (UTR) of target mRNA [9,10]. To date, more than 1000 miRNAs have been identified in human, and decades of them are differentially altered in almost all kinds of cancer. miR-143 and miR-145, transcribed from a putative cluster on chromosome 5q33, are coordinately expressed in a variety of cell lines and cancer tissues [11]. Previous studies showed that the 2 miRNAs were downregulated in BC, inhibiting cell proliferation, migration and invasion [12–14]. Accordingly, miR-143 and miR-145 were considered as tumour suppressors, and their dysregulation was recognized as an early event in malignant transformation [15,16].

Single nucleotide polymorphisms (SNPs) in the gene promoter region were demonstrated to be modulators of bladder cancer risk [17–19]. Recently, genetic polymorphisms in the promoter of miR-143/145 cluster have been reported to be related to the susceptibility of colorectal cancer [20], prostate cancer [21] and cervical squamous cell carcinoma [22]. However, no study has been done to investigate the association of SNPs in the promoter region of miR-143/145 with BC risk. In this study, a potentially functional rs353293 G/A was analyzed in a case-control study and luciferase activity was also examined *in vitro*.

## Materials and Methods

### Study population

A hospital-based case control study was performed in a total of 333 bladder cancer patients and 536 controls. All the cases were recruited from the affiliated hospital and affiliated northwest hospital of Youjiang Medical College for Nationalities between January 2010 and March 2015. Cases were newly diagnosed subjects with histological confirmation. Clinical data including tumor stage and grade were abstracted from medical record. Patients were excluded if they had a self-reported family history of cancer, radiotherapy and/or chemotherapy. The mean age (± standard deviation, SD) of the cases was 59.4 ± 11.6 years, and the male: female ratio in cases is about 3:2. Controls were healthy volunteers who came to the hospital for physical examination and individually matched to cases for age, gender, ethnicity, and geographic region. This study was approved by the institutional review board of the Youjiang Medical College for Nationalities and all participants provided written informed consent.

### Genotyping

About 3 ml ethylenediaminetetraacetic acid-anticoagulated peripheral blood was drawn from each participant. Genomic DNA was isolated using a phenol/chloroform extraction technique. Genotyping of the rs353293 in the promoter of miR-143/145 was done using the TaqMan assay (Applied Biosystems). To validate the genotyping results, about 5% of the samples were randomly selected for Sanger sequencing, and the results were 100% concordant.

### Plasmid construction of luciferase reporter genes

The promoter region of miR-143/145 containing rs353293G was synthesized by PCR amplification using primers described previously [22]. The PCR products were purified, extracted, and cloned into pGL3-basic vector (Promega, Madison, WI). The rs353293G was mutated into rs353293A using a QuickChange Site-Directed Mutagenesis kit (Stratagene, La Jolla, CA). The mutagenic primers were as follows: CCTGCTTCATGTTCTCACCCAC CCGGTGCC (forward), and GGCACCGGGTGGGTGAGAACATGAAGCAGG (reverse). Sanger sequencing was performed to confirm the orientation and integrity of the insert in the plasmid.

### Transient transfection and report assay

HeLa, RT4, and T24 were cultured in Dulbecco’s modified Eagle’s medium supplemented with 10% fetal bovine serum in 5% CO_2_ at 37°C. For transient transfection, all cells were seeded onto 24-well plates, and transfected with 1μg pGL3 constructs using Lipofectin 2000 (Life Technologies). As an internal control, all plasmids were cotransfected with 0.02μg pRL-SV40, which expressed *Renilla* luciferase (Promega Corporation). The pGL3-basic (empty vector) was served as a negative control. At 48 h post-transfection, the rs353293G and rs353293A promoter activities were determined by the Dual-Luciferase Assay System (Promega Corporation), and normalized against the internal control activity of *Renilla* luciferase. Each experiment was done in triplicate.

### Statistical analysis

Mean ages with standard deviations and frequencies of the basic characteristics were calculated. The distributions of age and gender between cases and controls were compared by using the Student’s *t* test or χ^2^ test. Hardy-Weinberg equilibrium was assessed by a goodness-of-fit χ^2^ test. The association between the rs353293 and risk of bladder cancer was estimated by computing odds ratio (OR) and their 95% confidence intervals (95% CI). The major homozygote and allele for the rs353293 were set as a reference. Adjusted odds ratios were computed for the potential confounding variables (age and gender) using multivariate logistic regression models. Differences of the relative expression of luciferase activity were determined by the Student’s *t* test. All statistical analyses were done using SPSS 11.0 software (Statistical Package for the Social Sciences, Chicago, IL). All tests were two sided, and *P* < 0.05 was considered to be significant.

## Results

### Characteristics of study subjects

Characteristics of the study population are shown in Table 1. We tested the association of demographic features in both cases and controls. There is no significant difference between cases and controls according to age and gender.

**Table 1:** Characteristics of the study population.

Variables	Controls (n = 536)	Patients with bladder cancer (n = 333)
Age (years, Mean ± SD)	58.0 (± 9.5)	59.4 (± 11.6)
Gender (%)		
Male	313 (58.4)	200 (60.1)
Female	223 (41.6)	133 (39.9)
Tumor stage (%)		
Ta/T1		137 (41.1)
T2-4		196 (58.9)
Tumor grade (%)		
High		180 (54.1)
Low		153 (45.9)

SD, standard deviation

### Association between the rs353293 polymorphism and risk of bladder cancer

Genotype frequencies of the rs353293 polymorphism and their association with risk of bladder cancer are shown in Table 2. The genotype distributions were in agreement with Hardy-Weinberg equilibrium in both cases and controls (*P* = 0.41 and 0.98). We calculated an adjusted odds ratio of 0.64 for the presence of either AA/AG genotypes (95% CI 0.46–0.90) and 0.64 (95% CI 0.47–0.87) for carrying at least one A allele in bladder cancer. That is, there was a 36% less chance of harboring the rs353293A allele in our bladder cancer group. Confirming this result in a general population study, and thereby validating the ‘A’ allele as a protective genetic marker, requires a large cohort analysis. We then compared patients with low grade tumors to those harboring higher grade bladder cancer, there was a similar result. The AA/AG genotypes and the A allele were less prevalent in patients with low grade tumors, compared to those harboring higher grade bladder cancers (adjusted OR = 0.53, 95% CI, 0.30–0.94, *P* = 0.03 and adjusted OR = 0.54, 95% CI, 0.32–0.92, *P* = 0.02, respectively). No significant association between the rs353293 polymorphism and tumor stage was detected (Table 3).

**Table 2:** Association between rs353293 G/A in the promoter region of miR-143/145 and risk of bladder cancer.

rs353293 G/A	Controls (n = 536) (%)	Bladder cancer (n = 333) (%)	Crude		Adjusted by age and gender	
			OR (95%CI)	*P* value	OR (95%CI) †	*P* value
GG	388 (72.4)	268 (80.5)	1.00		1.00	
AG/AA	148 (27.6)	65 (19.5)	0.64 (0.46–0.88)	0.006	0.64 (0.46–0.90)	0.008
G allele	912 (85.1)	599 (89.9)	1.00		1.00	
A allele	160 (14.9)	67 (10.1)	0.64 (0.47–0.86)	0.003	0.64 (0.47–0.87)	0.004

OR, odds ratio; CI, confidence interval

† OR was adjusted by age and gender.

**Table 3:** Stratified analyses of rs353293 G/A with clinical features of bladder cancer.

rs353293 G/A	Clinical features		Crude		Adjusted by age and gender	
			OR (95%CI)	*P* value	OR (95%CI)	*P* value
Tumor stage (%)	Ta/T1	T2-4				
GG	110 (80.3)	158 (80.6)	1.00		1.00	
AG/AA	27 (19.7)	38 (19.4)	0.98 (0.57–1.70)	0.94	0.97 (0.56–1.68)	0.91
G allele	247 (90.2%)	352 (89.8%)	1.00		1.00	
A allele	27 (9.8%)	40 (10.2%)	1.04 (0.62–1.74)	0.88	1.03 (0.62–1.73)	0.90
Tumor grade (%)	High	Low				
GG	137 (76.1)	131 (85.6)	1.00		1.00	
AG/AA	43 (23.9)	22 (14.4)	0.54 (0.30–0.94)	0.03	0.53 (0.30–0.94)	0.03
G allele	315 (87.5)	284 (92.8)	1.00		1.00	
A allele	45 (12.5)	22 (7.2)	0.54 (0.32–0.93)	0.02	0.54 (0.32–0.92)	0.02

### Effect of the rs353293 polymorphism on transcriptional activity

**Fig 1:**
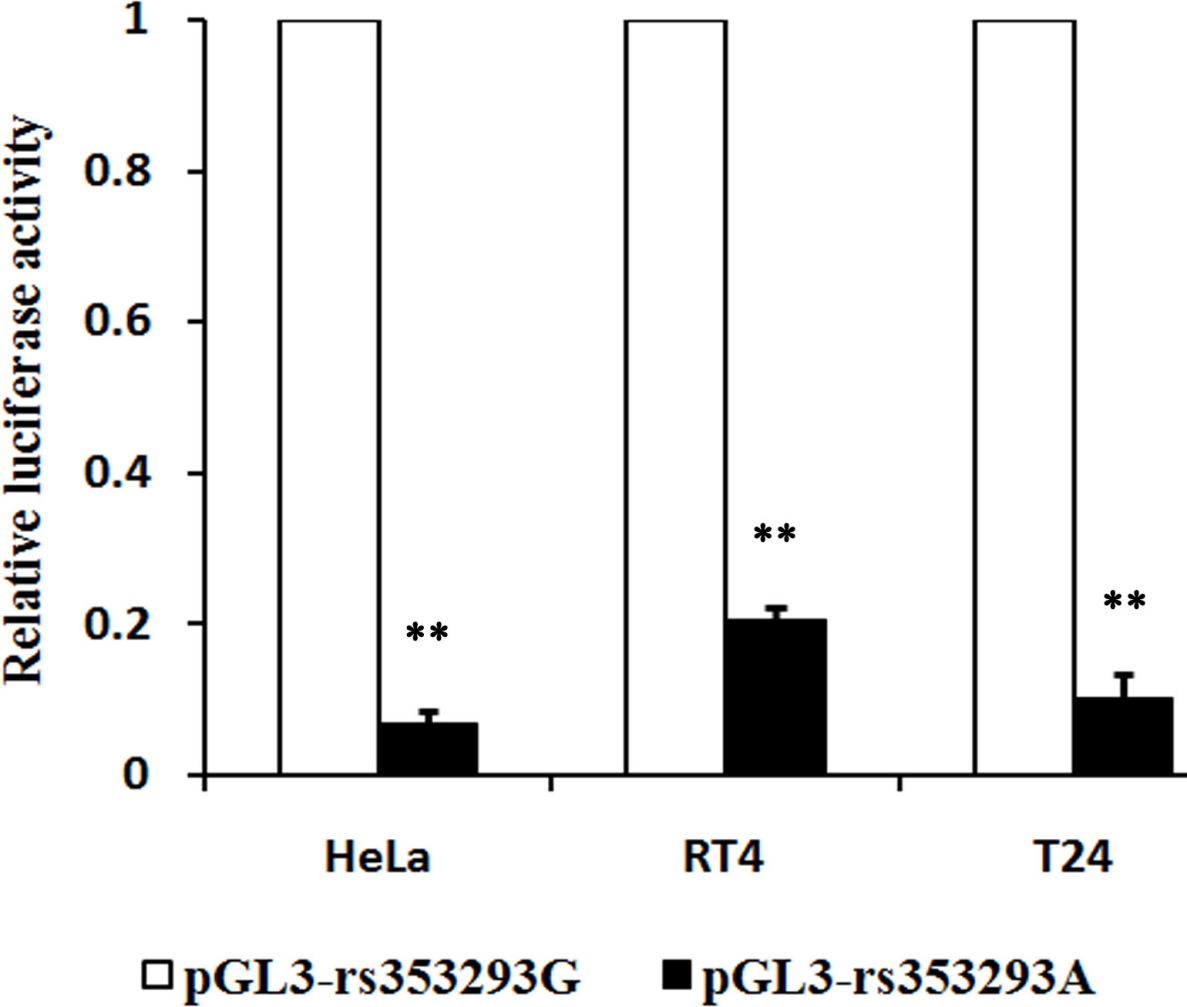
Effect of the rs353293 polymorphism in the promoter of miR-143/145 on the transcriptional activity. Compared with the rs353293G allele, the rs353293A allele had a reduced relative luciferase activity (**P < 0.001).

To evaluate the effect of the rs353293 polymorphism on transcriptional activity, we constructed luciferase reporter vectors (i.e., pGL3-rs353293G and pGL3-rs353293A). After transfected into HeLa, RT4, and T24 cells, relative luciferase activity was examined. As shown in Fig 1, the pGL3-rs353293A vector had a 93%, 80%, and 90% decreased activity compared with the pGL3-rs353293G in all three types of cell lines (*P* = 0.00002, 0.000001, and 0.001, respectively).

## Discussion

To the best of our knowledge, this is the first study to investigate the relationship of the rs353293 with bladder cancer risk. We found that the rs353293 AG/AA genotypes had a 0.64-fold decreased risk of bladder cancer, especially in patients with low-grade tumors. *In vitro* luciferase reporter analysis showed that rs353293A allele had a lower activity compared with the rs353293G allele. These findings suggest that the rs353293 may be used as a genetic biomarker for the etiology of bladder cancer.

miR-143 and miR-145 are clustered on the same chromosomal locus 5q33, which is a well-known fragile site in human genome. As representative anti-oncomiRs, the 2 miRNAs were highly co-downregulated in various carcinomas including cervical cancer, colorectal cancer, breast cancer, prostate cancer, and bladder cancer [12–14,23–27]. It is well-documented that miR-143/145 are involved in multiple cellular pathways underlying carcinogenesis. For instance, miR-143/145 can promote cell apoptosis and differentiation, and suppress cell proliferation, invasion and migration [28–31]. By targeting suppressor of cytokine signaling 7, miR-145 can promote interferon-β induction, resulting in the nuclear translocation of signal transducer and activator of transcription 3 [32]. Furthermore, several studies have demonstrated that miR-143/145 may constitute a marker to predict survival of bladder cancer, and exert antitumor effect in cancer therapy [33–36]. Intravesical administration of exogenous miRNA-145 can inhibit tumor growth and prolong animal survival [35]. Combination treatment with miR-143 and miR-145 has a synergistic effect on inhibition of cell growth [36].

It is evident that genetic variant in the gene promoter may influence individual’s susceptibility to bladder cancer [17–19]. As for SNPs in the promoter of miR-143/145, Li et al. analyzed 12 SNPs in this region and found that rs41291957, rs353292, rs353293, rs4705341, rs4705343, rs17796757, rs3733845 and rs3733846 were significantly associated with the risk of colorectal cancer [20]. Chu et al. reported that rs4705342TC/CC genotypes were associated with a significantly decreased risk of prostate cancer [21]. Liang et al. reported that rs4705343TC genotype was associated with an increased risk of cervical squamous cell carcinoma [22]. In this study, we found that the rs353293 AA/AG genotypes and the A allele were less prevalent in bladder cancer patients, indicating that the rs353293 A allele was a protective genetic marker for bladder cancer. It may be postulated that the polymorphism in the promoter of miR-143/145 alter the transcription. Consistent with this hypothesis, we found that the rs353293A exhibited a decreased luciferase activity. Further analysis got a surprising but interesting result, patients with the protective rs353293 genotypes tend to be diagnosed with higher grade lesions. There are some possible reasons for the result. The genetic protective effect may be very moderate in the development of bladder cancer. The severer the disease is, the less effect the polymorphism has. Additionally, we cannot rule out the possibility of chance occurring due to limited sample size, especially in stratification analysis. Future investigations with larger sample size should be instrumental in confirming our observations.

Although a strong association of the rs353293 with bladder risk was observed, some limitations still existed in this study. Study design is a key issue for a sound association study. We cannot exclude the possibility of selection bias of sample collection in the hospital-based case-control study. Population-based case-cohort study is needed to be done. Moreover, sample size enrolled in this study was moderate and all the participants were Chinese. Replication large-scale studies, therefore, are warranted to confirm this result in diverse ethnicities. As is known, environment exposure is a major risk factor for the development of bladder cancer. In this pilot study, we only take genetic factor into account. Further gene-environment interaction analysis should be essential.

In conclusion, this study suggests that the functional rs353293 polymorphism may be a useful biomarker to predict the risk of bladder cancer. These results have potential clinical implications. By genotyping this polymorphism, it may provide an additional option for improving the BC risk assessment in susceptible individuals. Additionally, this study provides insight into the possible mechanism of the promoter polymorphism in BC development.

## Abbreviations

OR: odds ratio
CI: confidence interval
BC: bladder cancer
UCC: urothelial cell carcinoma
3’ UTR: 3’ untranslated region
SNPs: single nucleotide polymorphisms
SD: standard deviation
PCR: polymerase chain reaction
